# Idiosyncratic Drug-Induced Liver Injury in a Patient With Metastatic Melanoma

**DOI:** 10.7759/cureus.89482

**Published:** 2025-08-06

**Authors:** Alessandra Ottley, Alison Thornton, Ramon Gil, Giezy Sardinas

**Affiliations:** 1 Osteopathic Medical School, Nova Southeastern University Dr. Kiran C. Patel College of Osteopathic Medicine, Davie, USA; 2 Graduate Medical Education, Hospital Corporation of America (HCA) Florida Northwest Hospital, Margate, USA; 3 Internal Medicine, Hospital Corporation of America (HCA) Florida Westside Hospital, Plantation, USA

**Keywords:** drug-induced liver injury (dili), hepatoxicity, immunotherapy-related adverse events, liver injury biomarkers, metastatic melanoma treatment

## Abstract

This is a case of drug-induced liver injury (DILI) in a 75-year-old male patient with a history of metastatic melanoma, who initially presented with a syncopal episode causing a fall. Following stabilization in the emergency department (ED), the patient was found to have bilateral subdural hematomas, and later an MRI showed evidence of metastatic lesions in the brain with hemorrhagic conversion. These findings led to a prolonged inpatient stay in the intensive care unit and eventual development of pneumonitis, which was subsequently treated with hepatotoxic antibiotics despite initial labs showing mildly elevated liver enzymes. This multidrug regimen and coexisting immunotherapy for metastatic melanoma led to drug-induced liver toxicity, a diagnosis that resulted in decreased cognitive function and an eventual decision to place the patient in hospice. While the patient eventually made a full recovery, this case report emphasizes the importance of maintaining a high index of suspicion for drug-induced liver toxicity in patients with risk factors such as receiving immunotherapy. DILI remains a serious complication in patients receiving immunotherapy, especially when combined with hepatotoxic antibiotics. Due to its difficult identification and frequent asymptomatic presentation, early recognition and intervention play a key role in preventing this sequelae.

## Introduction

Drug-induced liver injury (DILI) is defined as injury or disease of the liver caused by medications, herbs, or other toxic supplements. It is the most common cause of acute liver failure in the United States [1]. DILI can be further broken down into two types. Intrinsic DILI is defined as varying levels of hepatotoxicity that can occur in any individual, most commonly due to drugs that are administered at high doses, such as acetaminophen. Idiosyncratic DILI is hepatotoxicity in susceptible individuals; this form of DILI is less dose-dependent and more varied in its clinical presentation [2]. Diagnostic and therapeutic guidelines for intrinsic DILI are well established, as the presentation of acetaminophen toxicity is widely studied and easy to recognize [3-4]. However, idiosyncratic DILI is more difficult to identify and treat [5]. 

Most cases of idiosyncratic DILI present as asymptomatic or, in more severe cases, with fatigue, nausea, and jaundice [6-8]. The most common cause of DILI reported is antibiotics [7]. Most patients with DILI induced by antibiotic use have a favorable prognosis. However, those who present with jaundice have been noted to have a 10% risk of death due to liver failure [7]. Immunotherapy agents, particularly immune checkpoint inhibitors (ICIs), have been associated with idiosyncratic DILI [6]. Immunotherapy agents modulate the immune system in ways that can potentially lead to hepatotoxicity and make the patient more susceptible to DILI from antibiotics. Immunotherapy agents such as nivolumab have been proven to cause mild to moderate hepatitis [9]. While usually self-limited, nivolumab has led to severe hepatitis in 0.5% to 1.5% of patients [9]. Removal of the offending agent is the treatment of choice for DILI and, therefore, makes early identification of DILI crucial for preventing further complications such as acute liver failure leading to required transplantation [8]. This case report discusses a 75-year-old male patient with a history of metastatic melanoma receiving immunotherapy who presented with a syncopal episode and bilateral subdural hematomas. While inpatient, he developed DILI due to antibiotic use for hospital-acquired pneumonia. This case highlights the necessity of monitoring liver enzymes in patients who are at high risk for DILI.

## Case presentation

**Figure 1:**
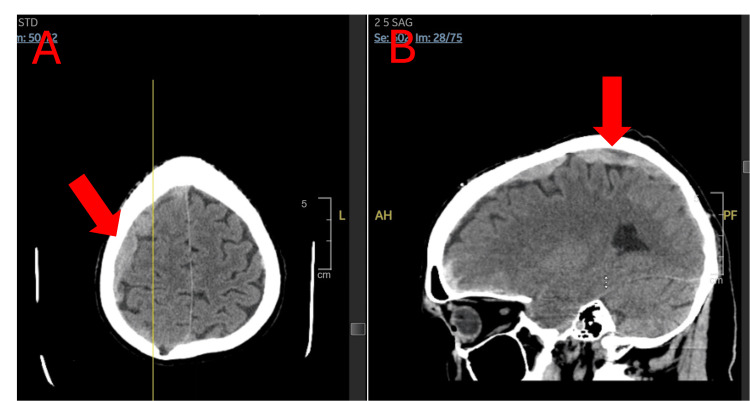
Initial CT of brain without contrast on admission displaying an anterior cranial fossa, suprasellar, bilateral sylvian fissure, and prepontine cistern subarachnoid hemorrhage in (a) transverse and (b) sagittal views.

**Figure 2:**
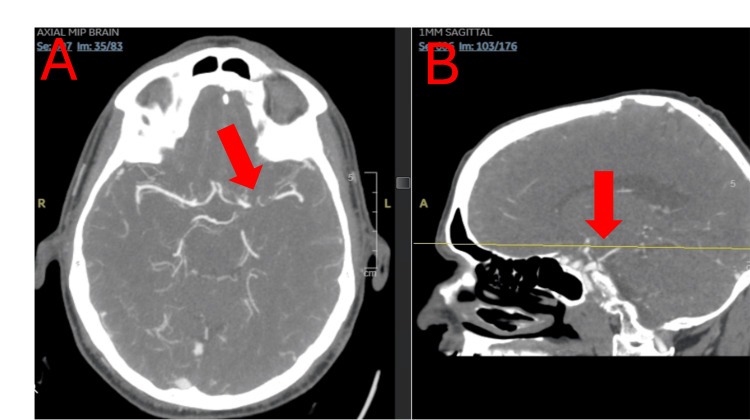
CT angiography without contrast displays bilateral anterior cerebral artery vasospasm (a) transverse; (b) sagittal views

This is a 75-year-old male patient with a past medical history of partial small bowel resection, hypertension, diabetes mellitus, hyperlipidemia, and stage 4 metastatic melanoma currently receiving immunotherapy for one month with Keytruda, a programmed cell death protein 1 (PD-1) inhibitor, who presented to the emergency department (ED) of a South Florida hospital with a chief complaint of a syncopal episode. While at work, the patient became lightheaded, leading to an unwitnessed fall, which resulted in a posterior head injury. The patient’s most recent immunotherapy session was the day prior to this hospital admission. In the ED, the patient was not following commands and was not oriented to place or time. His last known well time was two hours before his arrival at the ED. His National Institutes of Health Stroke Scale/Score (NIHSS) score was 13, meaning a moderate stroke likely occurred. The patient’s vital signs were as follows: temperature: 97.9 Fahrenheit, heart rate: 88 beats per minute, respiratory rate: 18 breaths per minute, blood pressure: 160/84 mmHg, mean arterial pressure: 109 mmHg, oxygen saturation: 100%, height: 5’6”, weight: 81.82 kg, body mass index: 29.1 kg/m². The patient's labs were drawn and are represented in Table 1. On physical examination, the patient had an altered mental status, was unable to follow commands, was responsive to painful stimuli, and spontaneously opened his eyes. Due to the head injury, the patient underwent a CT scan of the brain without contrast, showing an anterior cranial fossa, suprasellar, bilateral sylvian fissure, and prepontine cistern subarachnoid hemorrhage (SAH) (Figure 1). CT angiography without contrast showed bilateral anterior cerebral artery vasospasm (Figure 2). In the ED, he received fentanyl citrate 25 mcg and lorazepam 0.5 mg for pain control and sedation. He was admitted to the intensive care unit for further management.

**Table 1:** The patient’s laboratory workup on admission

Test name	Lab value	Reference range
Sodium	130	135-145 mmol/L
Potassium	4.9	3.5-5.2 mmol/L
Glucose	158	70-11 mg/dL
Aspartate aminotransferase	43	10-40 U/L
Alanine aminotransferase	35	10-60 U/L
Alkaline phosphatase	139	20-120 U/L
Total protein	7.5	5.5-8.7 g/dL
Albumin	3.9	3.2-5 g/dL
White blood cell	14.5	4.0-10.5 x 10^3^ /uL
Absolute neutrophils	5.45	1.56-6.13 x 10^3^/uL
Monocytes %	4	5.3-12.2%
Lymphocytes %	34	21.8 - 53.1

**Figure 3:**
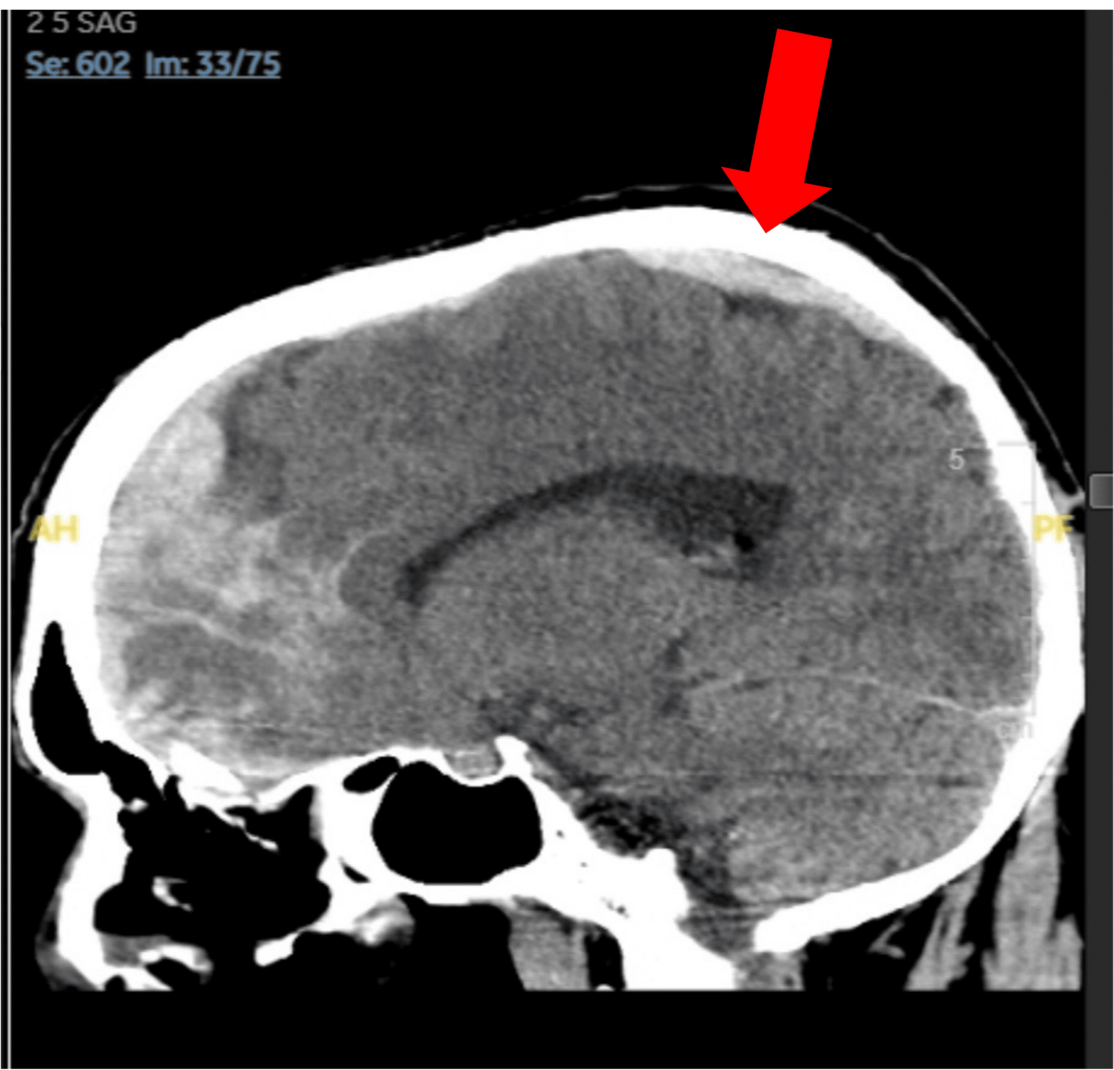
A CT scan of the brain without contrast, six hours after the initial CT, displaying worsening subarachnoid hemorrhage with developing intraparenchymal hemorrhage

On the second day of admission, the patient’s NIHSS score was five, still in the range of a moderate stroke. The patient was still unable to follow commands, was agitated and confused, with no speech deficits, and moved all extremities. A repeat CT brain without contrast was performed six hours after the initial CT (Figure 3) and showed worsening SAH with developing intraparenchymal hemorrhage (IPH). The patient was started on nimodipine, a Cardene drip, and 3% normal saline at 20 c/hr to assist with cerebral edema and an insulin drip. 

**Figure 4:**
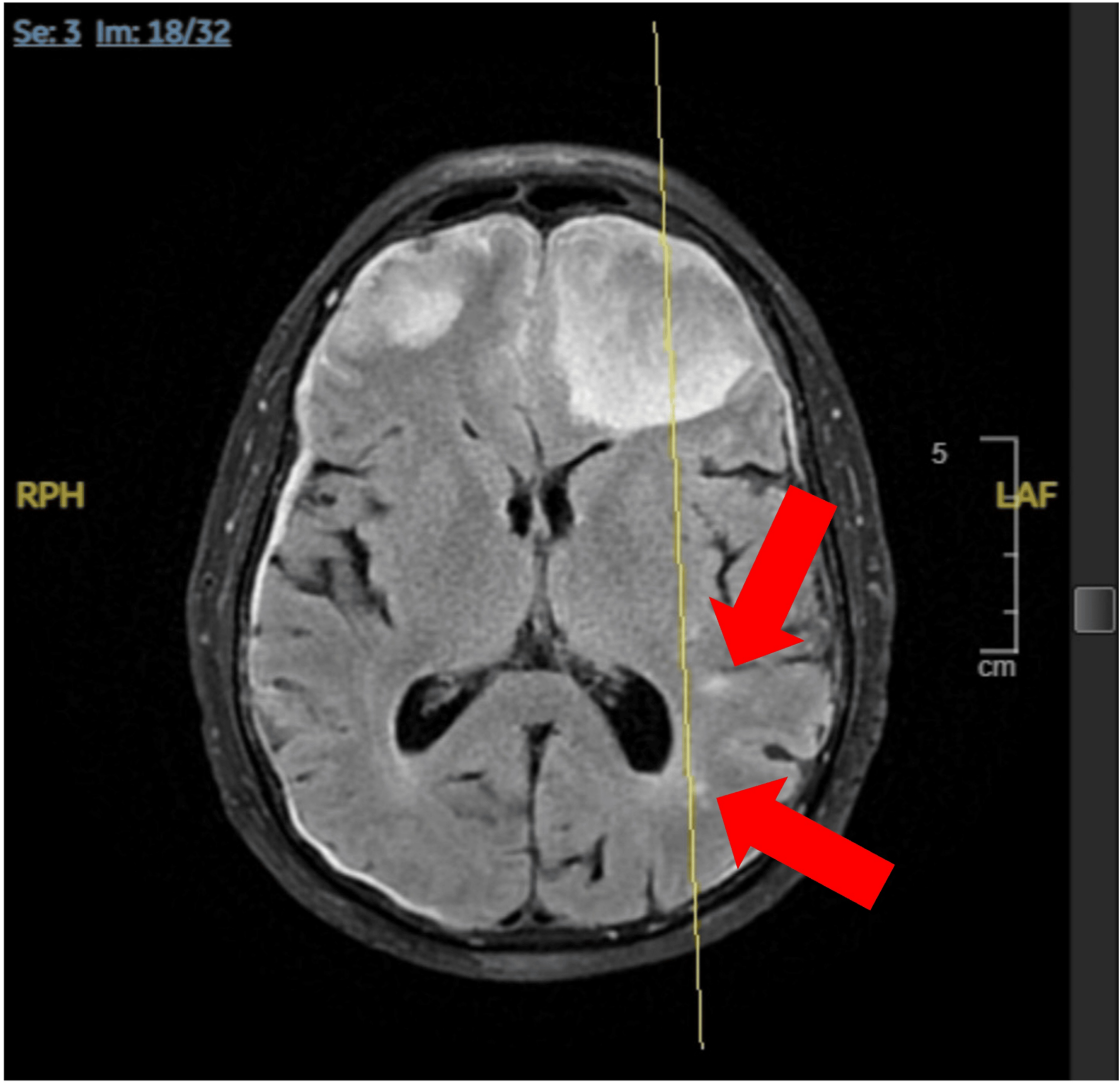
MRI of the brain displaying numerous punctate foci of dural-based nodular enhancement predominantly along the frontal convexities, suspicious for pachymeningeal spread of metastatic disease

On the third day of admission, an MRI of the brain showed numerous punctate foci of dural-based nodular enhancement predominantly along the frontal convexities, suspicious for pachymeningeal spread of metastatic disease (Figure 4). The patient was started on stress dose steroids (Decadron 4 mg every six hours) given the metastatic prognosis and history of underlying melanoma. 

On day four of admission, a chest X-ray revealed developing bilateral infiltrates and possible infectious pneumonitis, which was recommended by pulmonology to be followed with daily chest X-rays until resolved. An electrocardiogram (EKG) showed new-onset atrial fibrillation with rapid ventricular response and required an amiodarone 150 mg bolus followed by a continuous drip. 

Over the next three days, the patient had no acute events.

On day eight of admission, the patient was found to have worsening pneumonitis, and an antibiotic regimen was started, including vancomycin, cefepime, azithromycin, and augmentin, at the recommendation of the Infectious Diseases team. Alkaline phosphatase at the time was elevated at 157 U/L (normal 20-120 U/L). 

**Figure 5:**
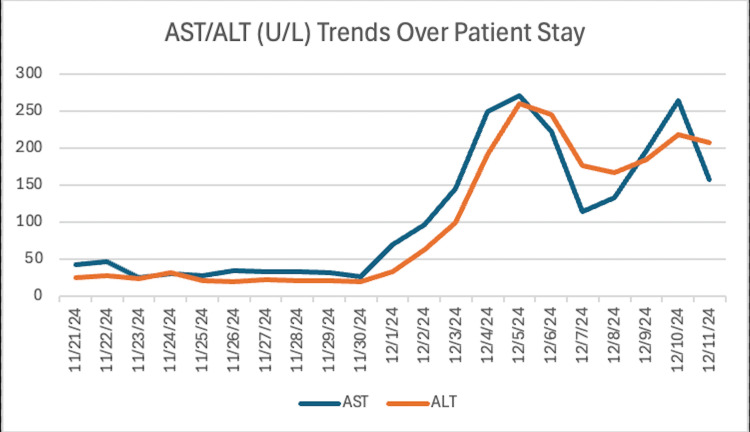
Patient AST/ALT (U/L) levels over inpatient stay showed a steep increase on day 10, peaking on day 15 and day 20. AST: aspartate aminotransferase; ALT: alanine aminotransferase

**Figure 6:**
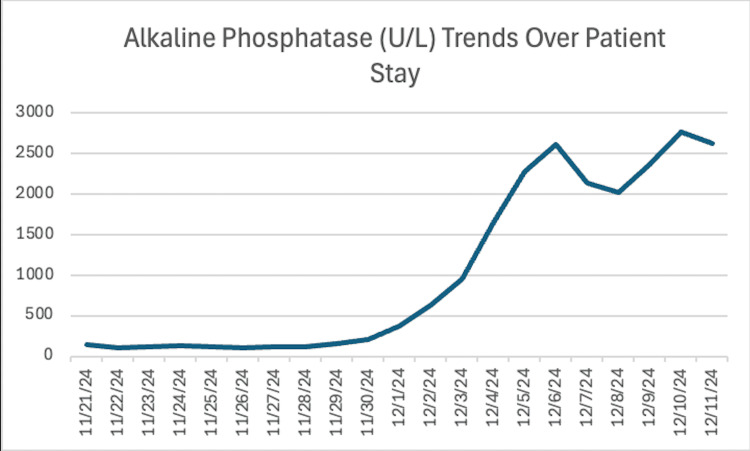
The patient's alkaline phosphatase (U/L) levels over the inpatient stay showed a steep increase on day 12, peaking on day 16 and day 20.

Over the next four days, the patient had significant improvement. The patient was examined sitting up in bed, oriented to place, person, and time, and was able to eat unassisted. However, liver enzymes continued to rise (Figures 5, 6).

On days 13 and 14, the patient presented with agitation and fever and was started on cefdinir/doxycycline for two days, in addition to his previous antibiotic regimen. Liver enzymes also continued to rise, and ursodiol was begun at the recommendation of gastroenterology. 

On day 15, magnetic resonance cholangiopancreatography (MRCP) was recommended due to persistently elevated liver enzymes, likely due to multiple hepatotoxic medications. MRCP showed several cystic foci concerning for branch intraductal papillary mucinous neoplasm (IPMN), and a liver biopsy was recommended. 

Over the next four days, the patient had no acute events, but liver enzymes continued to worsen. 

**Figure 7:**
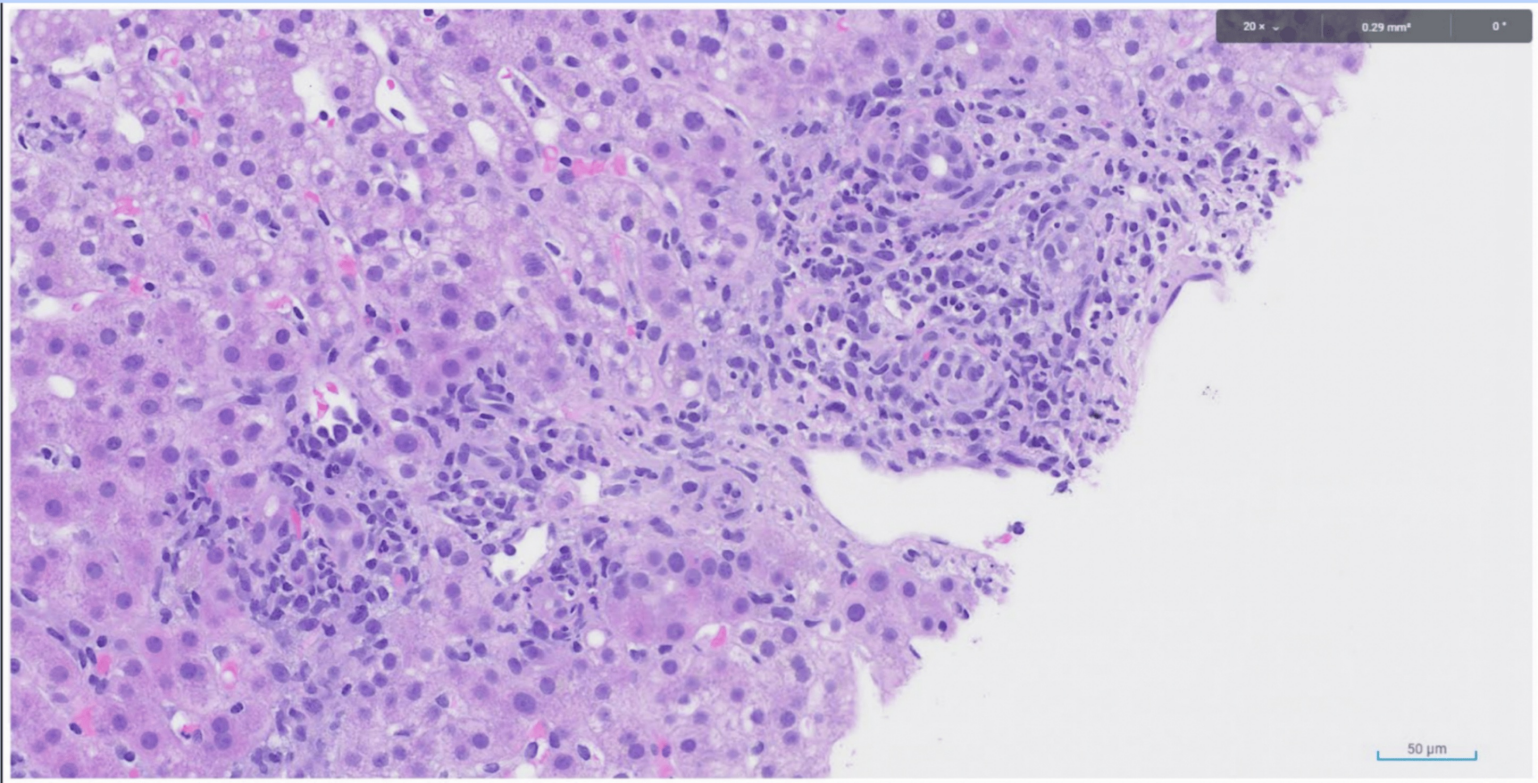
Liver biopsies displaying portal and lobular inflammation with bile duct and hepatocellular injury

**Figure 8:**
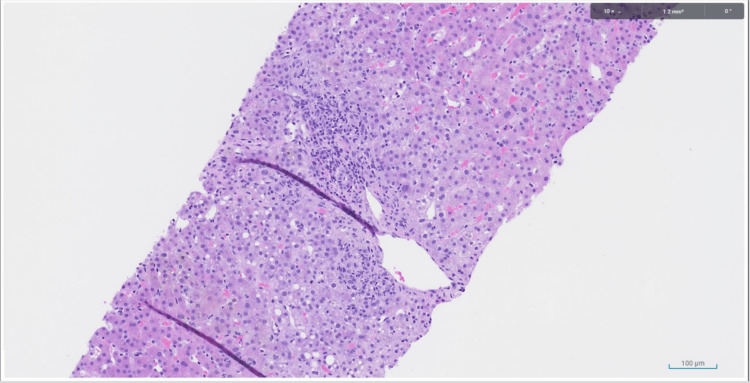
Liver biopsies displaying portal and lobular inflammation with bile duct and hepatocellular injury

On day 20, a liver biopsy revealed portal and lobular inflammation with bile duct and hepatocellular injury (Figures 7, 8), indicating that DILI was likely.

Outcome and follow-up

Due to the coexisting immunotherapy for metastatic melanoma occurring independently and the persistent elevation of liver enzymes, the patient and their husband decided that the patient would not benefit from a continued stay in the hospital. It was determined that the patient would discontinue all hospital medications and be discharged to home hospice. Three weeks after discharge, the patient’s cognition improved to baseline, and he continued his chemotherapy regimen. Upon completion of his scheduled chemotherapy regimen, the patient's oncology follow-up resulted in a cancer-free diagnosis.

## Discussion

This case report emphasizes the importance of monitoring liver enzymes in patients who are at risk for idiosyncratic DILI, particularly those who are concurrently receiving immunotherapy. In this patient, the past medical history of metastatic melanoma and concurrent treatment with immunotherapy placed them at a higher risk for DILI. Due to the clinical presentation of altered mental status and coexisting subdural hematomas, the patient had a prolonged hospital stay and developed pneumonia while in the hospital. The pneumonia was subsequently treated with azithromycin and amoxicillin/clavulanate, which are known to be hepatotoxic, on hospital day 10. These antibiotics, along with the patient’s prior month of immunotherapy treatments, accelerated the decompensation into DILI and eventual liver failure. Although the patient was not receiving immunotherapy during their hospital stay, the risk of DILI remained high. This is because immune-related adverse effects of ICIs, like Keytruda, can occur within days or weeks of beginning treatment or even months later. Liver enzymes began to steadily increase on hospital day 11. The diagnosis of DILI was made on hospital day 20 when a liver biopsy showed portal and lobular inflammation with bile duct and hepatocellular injury.

Few cases in the literature have made the correlation between certain medications and DILI, specifically idiosyncratic liver injury. In one report, azithromycin and amoxicillin/clavulanate were named as two of the most common hepatotoxic medications [10]. This patient was taking both of these medications throughout his 21-day hospital admission. The pneumonia complication required medical management with azithromycin and amoxicillin/clavulanate and put the patient at an increased risk of idiosyncratic DILI. What distinguishes our patient from these typical cases is the acute onset of the DILI. In most cases, idiosyncratic DILI presents weeks to months after initiation of medication, but in our patient, the process was much more acute, accelerating to DILI and then acute liver failure within 11 days of medication initiation. It is in the opinion of the authors that the acceleration was likely due to the patient’s history of metastatic melanoma, for which he was receiving immunotherapy.

On admission, the patient presented with mildly elevated liver function tests, likely due to their immunotherapy treatment, placing them at an elevated risk of developing liver failure. This risk was further increased with the use of pneumonia medications in the hospital. One study found that immunotherapy agents, namely ICIs, have led to higher reports of liver toxicity [11]. The adverse effects related to ICIs stem from the unregulated immune response and subsequent increase in T-cell-specific immune responses [11]. Our patient, with their metastatic melanoma diagnosis being treated with immunotherapy, was therefore likely to have already been at an increased risk of idiosyncratic DILI before their hospital admission. The subsequent pneumonia diagnosis and treatment likely further accelerated the process into DILI with the additional antibiotics to treat the pneumonia [10].

## Conclusions

This case displays the importance of maintaining a high index of suspicion for DILI in susceptible patients. It is crucial to take a thorough past medical history in every patient, especially patients who have a history of cancer and chemotherapy and/or immunotherapy treatment. Not only knowing that they are receiving chemotherapy or immunotherapy, but also which one they are receiving. DILI can be asymptomatic. In more serious cases, DILI can present with fatigue, nausea, and jaundice. Consistent monitoring of liver enzymes and prompt action when they become elevated is important for patient outcomes. Due to its difficult identification, it is important that a multidisciplinary approach be taken in possibly affected patients. Education should also be provided to patients partaking in immunotherapy for possible red flags of DILI and instructions on when to seek care. Early identification and intervention of idiosyncratic DILI are crucial to improve outcomes. When identified, the offending drug or drugs should be discontinued, and liver function tests should be continuously monitored until they return to baseline. 
